# Camphor in Ill-Conditioned Wounds

**Published:** 1877-01

**Authors:** 


### CAMPHOR IN ILL-CONDITIONED WOUNDS.

Under the name "phenicated camphor," Dr. Saulez describes (*Bull. de Therap.*, August 30) a combination of carbolic acid and camphor, which exerts a remarkable influence on wounds of an unhealthy character, as proved by him in the Romarantin Hospital. The acid will take up a large quantity of camphor, but Dr. Saulez ordinarily employes a solution of two grammes and a half of the powder in one of the acid (nine grammes to one of alcohol), which produces a pale yellow oleaginous liquid, in which the disagreeable odor of the acid is no longer to be recognized. It is not miscible with water or glycerine, and in employing it we have first to emulsify it by the addition of a twentieth part of the phenicated camphor either to olive oil or infusion of saporiana.

